# The effect of an apple polyphenol extract rich in epicatechin and flavan-3-ol oligomers on brachial artery flow-mediated vasodilatory function in volunteers with elevated blood pressure

**DOI:** 10.1186/s12937-017-0291-0

**Published:** 2017-10-27

**Authors:** Maria Saarenhovi, Pia Salo, Mika Scheinin, Jussi Lehto, Zsófia Lovró, Kirsti Tiihonen, Markus J. Lehtinen, Jouni Junnila, Oliver Hasselwander, Anneli Tarpila, Olli T. Raitakari

**Affiliations:** 1 Research Centre of Applied and Preventive Cardiovascular Medicine, University of Turku, Turku, Finland; 2 Department of Clinical Physiology and Nuclear Medicine, University of Turku and Turku University Hospital, P.O. Box 52, FI-20521 Turku, Finland; 3 Clinical Research Services Turku (CRST), University of Turku, and Unit of Clinical Pharmacology, Turku University Hospital, Turku, Finland; 4 DuPont, Nutrition and Health, Kantvik, Finland; 5 4Pharma Ltd, Turku, Finland; 6 DuPont, Nutrition and Health, Reigate, UK

**Keywords:** Epicatechin, Flavonoids, Flavanol-3-ols, Endothelial function, Vasodilatory function

## Abstract

**Background:**

The primary aim of this study was to test the hypothesis that an orally ingested apple polyphenol extract rich in epicatechin and flavan-3-ol oligomers improves endothelium-dependent brachial artery flow-mediated vasodilatation (FMD) in volunteers with borderline hypertension. The secondary aim of the study was to test whether the investigational product would improve endothelium-independent nitrate-mediated vasodilatation (NMD).

**Methods:**

This was a single centre, repeated-dose, double-blind, placebo-controlled, crossover study in 60 otherwise healthy subjects (26 men, 34 women; aged 40-65 years) with borderline hypertension (blood pressure 130-139/85-89 mmHg) or unmedicated mild hypertension (blood pressure 140-165/90-95 mmHg). The subjects were randomised to receive placebo or the apple polyphenol extract to provide a daily dose of 100 mg epicatechin for 4 weeks, followed by a four to five-week wash-out period, and then 4 weeks intake of the product that they did not receive during the first treatment period. FMD and NMD of the left brachial artery were investigated with ultrasonography at the start and end of both treatment periods, and the per cent increase of the arterial diameter (FMD% and NMD%) was calculated.

**Results:**

With the apple extract treatment, a significant acute improvement was detected in the mean change of maximum FMD% at the first visit 1.16 (*p* = 0.04, 95% CI: 0.04; 2.28), last visit 1.37 (*p* = 0.02, 95% CI: 0.22; 2.52) and for both visits combined 1.29 (*p* < 0.01, 95% CI: 0.40; 2.18). However, such improvement was not statistically significant when apple extract was compared with placebo. The overall long-term effect of apple extract on FMD% was not different from placebo. No statistically significant differences between the apple extract and placebo treatments were observed for endothelium-independent NMD.

**Conclusions:**

A significant acute improvement in maximum FMD% with apple extract administration was found. However, superiority of apple extract over placebo was not statistically significant in our study subjects with borderline hypertension or mild hypertension. The study raised no safety concerns regarding the daily administration of an apple polyphenol extract rich in epicatechin.

**Trial registration:**

The trial is registered at http://clinicaltrials.gov (identifier: NCT01690676). Registered 25th May 2012.

## Background

Flavonoids are phenolic secondary plant metabolites which have been further subclassified as flavonols, flavones, isoflavones, flavanones, anthocyanidins, and flavan-3-ols. Flavan-3-ols comprise monomeric flavan-3-ols, or ‘catechins’, such as epicatechin as well as oligomers and polymers of catechins called proanthocyanidins. Previous experimental studies have indicated that epicatechin can exert cardioprotective actions, which may involve endothelial nitric oxide synthase (eNOS) -mediated nitric oxide production in endothelial cells. Treatment of cultured endothelial cells with epicatechin activates eNOS [1, 2]. Several clinical studies [3–6] have demonstrated that cocoa, which is a rich source of flavan-3-ols, improves endothelial function in man. Pure epicatechin has also been shown to have an acute beneficial effect on endothelial function [3], and a non-significant tendency for improved flow-mediated vasodilatation (FMD) has been reported following supplementation with 100 mg epicatechin for 4 weeks [7]. Thus, it has been suggested that ingestion of epicatechin is at least partially responsible for the reported vascular effects observed after consumption of cocoa [8].

Hypertension is a major risk factor for atherosclerosis. It has been estimated that the risk of coronary heart disease is doubled with each blood pressure (BP) increment of 20/10 mmHg (systolic/diastolic), beginning at 115/75 mmHg. Elevated BP may interfere with the integrity of arterial endothelial cells, causing endothelial dysfunction [9]. Endothelial dysfunction is one of the earliest changes in arteries associated with the process of atherosclerosis. A non-invasive ultrasound technique to evaluate endothelium-dependent FMD of the brachial artery has been used in clinical studies on endothelial function. Impaired endothelial function, assessed with the FMD method, was associated with the risk for cardiovascular events in middle-aged adults, also after adjustment for age, sex, diabetes mellitus, cigarette smoking, systolic BP, high-density lipoprotein (HDL) cholesterol, low-density lipoprotein (LDL) cholesterol, triglycerides, heart rate (HR), statin use, and BP medication use [10]. Brachial FMD responses have been in agreement with the results of tests carried out with invasive methods to assess coronary and brachial artery endothelial function [11]. Impaired brachial artery endothelial function is also associated with the prevalence and extent of coronary atherosclerosis [12]. In cross-sectional studies, BP levels have been shown to be negatively associated with FMD, and hypertensive subjects have been observed to have decreased FMD responses [13].

The primary aim of this study was to test the hypothesis that an orally ingested apple polyphenol extract rich in epicatechin and flavan-3-ol oligomers improves brachial artery endothelial function in volunteers with borderline hypertension. The secondary aim of the study was to test whether the investigational product would non-specifically improve the capacity for vasodilatation, as assessed with nitrate-mediated vasodilatation (NMD) responses. In addition, clinical and laboratory safety assessments were performed before and during the intervention.

## Methods

This was a single-centre, repeated-dose, double-blind, placebo-controlled, crossover study to evaluate the effect of an apple-derived polyphenol extract on endothelial function in otherwise healthy subjects with borderline hypertension or unmedicated mild hypertension.

### Participants

**Fig. 1:**
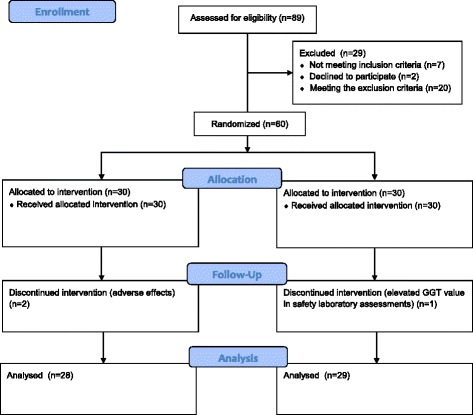
Study flow diagram

The study recruited 60 otherwise healthy male and female volunteer subjects, aged 40-65 years, with borderline hypertension or mild hypertension. A study flow diagram is shown in Fig. 1.

The inclusion criteria of the study called for the presence of borderline hypertension (BP 130-139/85-89 mmHg) [14] or mild hypertension (BP 140-165/90-95 mmHg) [15] at the screening visit (mean BP value of two measurements), otherwise good general health as ascertained by medical history, clinical laboratory assessments and physical examination, and willingness to comply with the study procedures. Written informed consent was obtained from all the subjects prior to study participation. Exclusion criteria consisted of body mass index (BMI) >32 kg/m^2^, total serum cholesterol ≥8 mmol/l, any abnormal safety laboratory parameter or abnormal finding in the electrocardiogram (ECG) evaluated to be clinically significant, coronary artery disease, pregnancy or lactation, alcohol abuse as evaluated by medical history, laboratory determinations and an alcohol breath test, regular smoking, diabetes mellitus, apple allergy, use of lipid-lowering medication, regular use of any medication known or believed to affect endothelial function or blood vessel tone, high consumption of products containing flavonoids, habitual use of vitamin products containing single vitamins in strengths beyond the recommended daily intake, habitual use of herbal remedies, participation in any clinical trial during the previous 2 months and any other condition or medication that in the opinion of the investigator would interfere with the evaluation of the study results.

### Study design

**Fig. 2:**
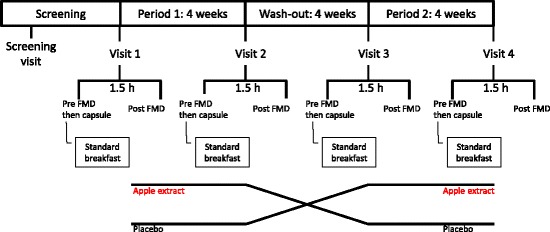
Study design. Screening visit was conducted within 30 days prior to the first study product administration. FMD, flow-mediated dilatation. Sequence: apple extract – placebo, sequence: placebo – apple extract

The subjects were randomized in a cross-over design to receive placebo or the apple polyphenol extract rich in epicatechin and flavan-3-ol oligomers for 4 weeks (“Treatment Period 1”), followed by a four to five-week wash-out period and finally 4 weeks of treatment with the product that they did not receive during the first Treatment Period of the study (“Treatment Period 2”). There were altogether five scheduled visits to the study site - one at screening (“Screening Visit”), and two visits in each Treatment Period. In the Treatment Periods 1 and 2 first visit was conducted when the investigational product intake was started (“Visit 1” in Treatment Period 1 and “Visit 3” in Treatment Period 2) and the second visit when the last dose was to be taken (“Visit 2” and “Visit 4”). For practical reasons, it was allowed that each subject could take the investigational product for 26-30 days in each Treatment Period. The study design is shown in Fig. 2.

Prior to all visits 10 h of fasting was required. Use of alcohol and strenuous physical exercise were forbidden for 24 h prior to all visits. Cigarette smoking or use of other nicotine products was not allowed for 10 h prior to each visit and during the visits. Otherwise the subjects were to follow their habitual diet and lifestyle during the entire study.

### Screening visit

A written informed consent was obtained. The subjects were interviewed for collection of demographic data (date of birth, age, race, sex, special diets) and information on substance use (use of nicotine, alcohol and caffeine, and drug abuse), use of epicatechin -containing foods and drinks and participation in other clinical trials. Body weight, height and BMI were recorded, blood samples were collected for haematology, clinical chemistry and serology after fasting for at least 10 h, a urine sample was collected for drug abuse and pregnancy tests, an alcohol breath test was performed, a standard physical examination was performed, medical history and current medical conditions were recorded, use of medications (including prescription and over-the-counter medications, dietary supplements, herbal products, micronutrients and vitamins) within two weeks prior to the screening visit was recorded, a 12-lead ECG was recorded in the supine position after 10 min rest, systolic and diastolic BP and HR were recorded twice from the right arm with 1-2 min interval in the sitting position after 5 min rest. Diet diaries with instructions were given to the subjects with an instruction to fill the diary on four successive days, after the Screening Visit and at the end of both Treatment Periods. A subject was invited to the Visit 1 if all of the inclusion criteria and none of the exclusion criteria were met. The study events in five different visits are shown in Table 1.

**Table 1:** Study events in five different visits

Study event	Screening visit	Visits 1 and 3 (first visit of both periods)	Visits 2 and 4 (last visit of both periods)
Informed consent	x		
Baseline characteristics	x		
Blood pressure, heart rate	x	x	x
Electrocardiogram	x		
Physical examination	x		
Laboratory determinations:			
Haematology	x		x
Clinical chemistry	x		x
Diet diary filling for 4 days^a^	x		
Start of study product intake		x	
Study product intake		x	x
Return of diaries			x
FMD twice, NMD once		x	x
Collecting of adverse events	x	x	x
Concomitant medications	x	x	x
Blood sampling for epicatechin and biomarker analyses prior to the morning dose and 2 h thereafter		x	x

^a^3 week-days, 1 weekend-day. First filling period after screening visit but before first treatment period. During treatment periods, filling on the 4th week of the treatment

FMD, flow mediated dilatation

NMD, nitrate mediated dilatation

### Investigational product

The apple polyphenol extract rich in epicatechin and flavan-3-ol oligomers had the following composition: high content of polyphenols (around 90% total polyphenols as catechin equivalents), specifically at minimum 30% (−)-epicatechin and at least 20% flavan-3-ol oligomers (degree of polymerisation (dp)2-dp7) and minor amounts of oligomers with dp > 7, other catechins and polyphenols. The apple extract was provided by Coressence Ltd. The source apples (Evesse™ Apples) for the extract have the botanical classification *Malus pumila* MILLER, more generally known as *Malus domestica* or the domestic modern apple. Evesse™ Apples are selected from the Herefordshire Pomona in the UK, a nineteenth Century catalogue of variety type / names of apples and pears known to have been grown in Herefordshire [16]. The apples are harvested at maturity in a period between August and November each year and either freeze dried and powdered or stored in controlled atmosphere and/or coldstores at chilled conditions (2-5 °C) until processed. Whole fresh apples are de-waxed by an ethanol wash then combined with a proportion of freeze dried apple granules and hot water. While continually stirring, the extraction mixture is rapidly cooled and enzymes are added to assist in the liberation of flavan-3-ols from the apple granule matrix. Denaturing of the enzymes by heating and filtration yields a sugar-rich syrup. Residual carbohydrate polymers are further degraded with enzymes before the sugar-rich intermediate extract is passed over an ion-exchange column to remove sugars. The apple polyphenol extract rich in epicatechin is precipitated using food-grade ethanol and dried. The apple extract is stable at ambient temperatures for at least 18 months from date of manufacturing. Epicatechin and flavan-3-ol content was determined by Reversed Phase HPLC.

In a study by Schroeter et al. [3] vascular response measured by the flow mediated dilation and pulse amplitude tonometry was improved with oral administration of 1 or 2 mg/kg bw of epicatechin in a small proof-of-concept study with healthy volunteers. Similarly, significant increases in vascular response were reported after oral administration of 1.5. mk/kg bw of epicatechin [17]. These studies provide rationale for a target dose of dietary supplement delivering 1-2 mg/kg bw epicatechin to improve vascular function. Thus, in this study the daily dose of 100 mg epicatechin was administrated.

The study product was given orally as a single dose of 330 mg/day, containing 100 mg of epicatechin, and encapsulated in hard gelatine capsule. The study subjects were instructed to take the study product preferably in the morning, with breakfast, approximately at 8 a.m. (between 6 and 10 a.m.). The first and the last study product dose of each treatment period were administered at the study site after a standard breakfast.

The placebo capsules had similar appearance as verum/epicatechin capsules but contained 335 mg Microcrystalline cellulose (MCC) (Tabulose Blanver, Florida, USA). Both placebo and verum capsules contained 3 mg Mg-Stearat. Epicatechin capsules were balanced with placebo capsules by adding 50 mg MCC.

### FMD and NMD responses

Endothelium-dependent and -independent vasodilatation of the left brachial artery was monitored with ultrasonography (Acuson Sequoia 512 mainframe instrument; Acuson, Mountain View, CA, USA) with a 13.0-MHz linear-array transducer. The method was based on the measurement of brachial arterial diameter at baseline and after a vasodilatory stimulus.

Brachial artery scans were obtained at rest and during reactive hyperaemia to study endothelium-dependent FMD. The luminal diameter of the artery was first measured at rest at a fixed distance from an anatomical marker at end-diastole, incident with the R wave on the ECG (3 measurements; the mean was used in the analyses). The artery was then occluded by inflating a blood pressure cuff placed around the forearm to a pressure of 250 mmHg. After 4.5 min, the cuff was deflated, causing increased blood flow in the artery, i.e. reactive hyperaemia. The arterial diameter was now recorded at 40, 60 and 80 s after cuff release, and dilatation of the artery from baseline was measured offline at end-diastole, incident with the R wave. From these data, the maximal FMD response was calculated as per cent increase of the arterial diameter from the baseline [18].

The pre and post FMD tests were performed on all Visits 1 to 4. The pre FMD test was performed at baseline, prior to study treatment administration, and the post FMD test was repeated 1.5 h after the administration. The timing of the second FMD measurement was based on a recent human bioavailability study showing that the time for maximum concentration (T_max_) of the active moiety (epicatechin and its metabolites: 3’methylepicatechin, 4’methylepicatechin, epicatechin sulfates and methyl-epicatechin sulfates) observed for a drink fortified with the same apple polyphenol extract rich in epicatechin and flavan-3-ol oligomers was at 1.0 -1.5 h [19].

Long-term changes in endothelial function were evaluated by comparing the pre FMD test values measured at the beginning and end of each Treatment Period. Acute changes in endothelial function were evaluated by comparing the pre and post FMD test values measured on the first visit of each treatment period (Visits 1 and 3). Additionally, acute changes in endothelial function were assessed by comparing the pre and post FMD test values measured on the last visit of each Treatment Period (visits 2 and 4), and taking into account the possible long-term change.

The NMD test was performed on all Visits (1-4), after the post FMD test. A new baseline measurement of the luminal diameter was performed 10 min after the postFMD test, and a dose of 1.25 mg of isosorbide dinitrate (Dinit, Leiras, Finland, 1.25 mg/dose; 1 spray) was administered sublingually, and the arterial diameter was measured 4 min later. From this measurement, the NMD% (per cent increase of the arterial diameter from the baseline) was calculated. Possible changes in vascular function were evaluated by comparing the two NMD values measured at the start and end of each Treatment Period and the two NMD values measured at the end of each Treatment Period.

Changes in systolic and diastolic BP values between the visits were also evaluated.

### Circulating biomarkers

Serum and plasma samples were collected for analysis of markers of inflammation, adhesion molecules and coagulation markers. These included soluble E-selectin (sE-selectin), soluble vascular cellular adhesion molecule-1 (sVCAM-1), soluble intercellular adhesion molecule-1 (sICAM-1), von Willebrand factor (vWF), plasminogen activator inhibitor-1 (PAI-1), asymmetric dimethylarginine (ADMA) and C-reactive protein (CRP) (sensitive assay). Concentrations of sE-selectin and ADMA were determined from serum samples, whereas the other markers were determined from plasma samples. The biomarkers were analyzed with commercial enzyme linked immunosorbent assay (ELISA) kits according to instructions provided by the manufacturers: ADMA (DLD Diagnostika Gmbh, Hamburg, Germany), vWf (RayBiotech, Norcross, GA, USA), sVCAM, sICAM-1, PAI-1, sE-selectin, CRP (Aushon Biosystem, Billerica, MA, USA). Each individual subject’s samples were analyzed on the same ELISA plate to reduce analytical variation. Biomarker samples were collected at the same time points as those collected for the determination of epicatechin concentrations, prior to and 2 h after dosing on each Visit.

Concentrations of epicatechin and its metabolites were analysed by IFR Extra Ltd. (www.ifrextra.co.uk) using liquid chromatography-tandem mass spectrometry, as described previously [20].

### Assessment of safety

At the screening visit, blood samples were collected for haematology, clinical chemistry and serology. Urine samples were also collected for drug abuse and pregnancy tests.

Systolic and diastolic BP and HR were recorded at all Visits. At the last visits of both Treatment Periods (Visits 2 and 4), blood samples were collected for safety determinations. Any clinically significant abnormal laboratory or other safety values were followed up.

Laboratory safety variables measured included haemoglobin, haematocrit, reticulocytes, mean corpuscular volume, mean corpuscular haemoglobin, leucocytes, differential blood count (neutrophils, lymphocytes, monocytes, eosinophils, basophils), plasma albumin, plasma bilirubin, plasma alanine aminotransferase, plasma aspartate aminotransferase, fasting plasma creatinine, plasma glutamyl transferase, plasma sodium, plasma potassium, fasting plasma calcium, fasting plasma urea, plasma uric acid, fasting plasma total cholesterol, fasting plasma HDL-cholesterol, fasting plasma LDL-cholesterol (calculated), fasting plasma triacylglycerols, fasting plasma glucose and plasma CRP.

Adverse events registered during the treatment periods were isolated occurences of nasopharyngitis, dyspepsia, headache, migraine, dizziness, ligament sprain and gammaglutamyltransferase increase, with no obvious association to either treatment.

### Assessment of dietary intake

Diet diary data were collected for the evaluation of the possible effects of diet on the study results. Dietary intakes were analysed three times using four-day food intake records; once after the Screening Visit and once during both Treatment Periods to estimate possible changes in the subject’s diet during the course of the interventions. A nutritionist checked the food records for accuracy at each occasion. The nutrient intakes were calculated with Micro-Nutrica software based on the Food and Nutrient Database of the Social Insurance Institution, Finland. The programme has been continuously updated with new foods and recipes in conjunction with the STRIP study at the Research Centre of Applied and Preventive Cardiovascular Medicine, University of Turku [21].

### Statistical analyses

Based on the results of previous studies [5, 22] a mean (SD) difference between the treatments in FMD% change from baseline of 1.0% (SD 2.5) was assumed. With 80% power (β = 0.20) and two-sided type I error risk of 5% (α = 0.05), 26 subjects per sequence was calculated to be needed. Assuming a 15% lost-to-follow-up rate, a total of 60 subjects were needed to be recruited for the study, i.e. 30 subjects per the cross-over treatment sequence.

The primary efficacy variable was the change from baseline in FMD%, including both the long-term and the acute response. Subjects who completed the both Treatment Periods and had a full set of FMD measurements were used in the evaluation of the primary hypothesis.

The primary efficacy variable (maximal FMD% response) was analyzed with two separate statistical models; one assessed the long-term change in the FMD response and one assessed the acute change. An analysis of covariance (ANCOVA) model was used to analyze the long-term effect based on the changes in FMD%. The model included baseline FMD% as covariate, and the main effects of cross-over sequence, Treatment Period and treatment as fixed effects and subject as random effect. The difference in the change of FMD% (apple extract - placebo) at end-of-period and a two-sided 95% confidence interval (CI) for the difference were estimated from the ANCOVA model using contrasts. Estimates of within-group changes were provided, as well.

The acute effects were analyzed using the changes in FMD% between the measurements on the first visits of both treatment periods (Visits 1 and 3). A similar model was applied in the statistical analysis as for the long-term effects. The acute changes were also characterized by comparing the changes in FMD% between the measurements on the last visits of both treatment periods (Visits 2 and 4).

In addition, as no long-term effects were detected, a Wallenstein-Fisher model was used to compare the acute changes in FMD% using both the first and the last visits of each period. This model included main effects of treatment, sequence, Treatment Period and Visit (within the Treatment period) as well as sequence by visit, period by visit and treatment by visit interaction effects as fixed factors. The secondary efficacy variables were the brachial NMD% test result, systolic and diastolic BP and the seven investigated biomarkers (sICAM-1, sVCAM-1, PAI-1, CRP, ADMA, vWf and sE-selectin). The analyses of the secondary variables were based on data from all subjects who completed both Treatment Periods and had full sets of measurements available.

A similar Wallenstein-Fisher model was applied for the NMD% as in the primary analysis. Similar ANCOVA models were used for systolic and diastolic BP as for the long-term effect in the primary FMD% analysis. For the biomarkers, both long-term and acute effects were evaluated. Long-term effects were evaluated with similar ANCOVA models as the FMD% and acute effects with similar Wallenstein-Fisher models as the FMD%. A natural logarithmic transformation was used for the following biomarkers: vWf, E-selectin, CRP, sVCAM-1 and PAI-1. This was done to normalize the distributions to be able to use analysis methods assuming normal distributions. The analysis results for these variables are presented in the log-scale.

All statistical analyses were performed and tables, figures and subject data listings were prepared with SAS software version 9.3 (SAS Institute Inc., Cary, NC, USA).

## Results

Sixty subjects were randomized into this study: 57 individuals completed the study and three discontinued prematurely because of adverse effects (migraine, dyspepsia and increased level of plasma gammaglutamyltransferase). The study Visits were between July 2012 and May 2013. Participants who completed both Treatment Periods and had full sets of measurements were used in the evaluation of the primary and secondary efficacy variables.

The mean age of the all-Caucasian study population was 55.3 years (SD 7.2, range 41-66 years). Twenty-six (43.3%) men and 34 (56.7%) women were included. The average BMI was 25.5 kg/m^2^ (SD 3.3). Demographic characteristics of the study subjects per treatment sequence are shown in Table 2. There were no significant differences between the groups.

**Table 2:** Demographic characteristics of study subjects

Variable (SD)	Apple extract – placebo sequence	Placebo- apple extract sequence
Age, y	55.6 (7.9)	54.9 (6.4)
Male sex, n	13	13
BMI, kg/m^2^	24.7 (3.0)	26.3 (3.5)
Systolic blood pressure, mm Hg^a^	148 (12)	150 (12)
Diastolic blood pressure, mm Hg^a^	91 (6.5)	93 (5.9)
LDL cholesterol, mmol/l	3.1 (0.9)	3.5 (0.8)
HDL cholesterol, mmol/l	3.1 (0.9)	3.5 (0.8)
Triglycerides, mmol/l	1.0 (0.3)	1.2 (0.7)
Glucose, mmol/l	5.4 (0.4)	5.5 (0.5)
C-reactive protein, mg/l	2.8 (2.0)	2.2 (1.2)
Alcohol consumption, n		
Non-drinker	2	5
Moderate consumption	28	25
Use of nicotine products, n		
Never used	22	22
History of use	5	6
Current use	3	2
Caffeine consumption, n		
Does not use caffeine	0	2
≤ 600 mg caffeine/day	27	22
> 600 mg caffeine/day Epicatechin intake (mg)	3 8.2 (5.8)	6 9.2 (5.1)

^a^Screening visit

According to the food diaries no differences in daily dietary intake of macronutrients, vitamins or major food groups known to contain flavonoids (e.g. apples, tea, cocoa, red wine) were seen between the Screening Visit records or those of the Treatment Periods. The mean average daily intake of epicatechin according to interviews was 8.7 mg (SD 5.4, range 1-20 mg) at Screening Visit.

### Primary efficacy parameters

#### Acute effect of apple extract on maximal FMD%

**Fig. 3:**
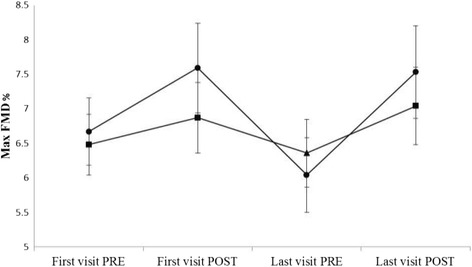
The maximal FMD% by the treatment periods after apple extract and placebo administration. The figure shows the mean values and S.E. for each FMD measurements that were taken before and after the dosing during the first and last treatment visits. FMD, flow-mediated dilatation

**Fig. 4:**
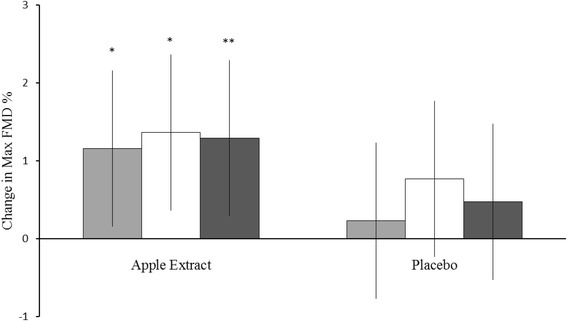
The acute changes from baseline in maximal FMD% within visits and for the visits combined. The figure shows mean change in FMD% from baseline. The error bars represent 95% CI. *p < 0.05 and **p < 005. FMD, flow-mediated dilatation. Test for difference between groups in combined visits *p* = 0.181. Test for difference epicatechin vs baseline: 1. visits *p* = 0.043, 2. visits *p* = 0.02, combined *p* = 0.005

Significant acute mean improvements were detected in maximum FMD% with apple extract at the first visits of the Treatment Periods (1.2%, 95% CI from 0.0% to 2.3%, *p* = 0.043), at the last visits of the Treatment Periods (1.4%, 95% CI from 0.2% to 2.5%, *p* = 0.02) and for all the Visits 1-4 combined (1.3%, 95% CI from 0.4% to 2.2%, *p* = 0.005). The corresponding acute effects with placebo were insignificant mean increases (Visits 1 and 3 0.2%, 95% CI from −0.9% to 1.4%; Visits 2 and 4 0.8%, 95% CI from −0.4% to 1.9%, and Visits 1-4 0.5, 95% CI from −0.4% to 1.4%). However, superiority of apple extract over placebo was not shown (test for difference between treatments on Visits 1 and 3 *p* = 0.224, on Visits 2 and 4 *p* = 0.329, and Visits 1-4 combined, *p* = 0.181). In addition, the Treatment Periods in this cross-over trial were statistically significantly different in the model including both visits within the periods (*p* = 0.045). The changes in maximal FMD% over Treatment Periods after apple extract and placebo administration are presented in Fig. 3 and acute changes (combined pre to post FMD test) in FMD% in Fig. 4.

#### Long-term effect of apple extract on maximal FMD%

The change from baseline FMD% (Visits 1 and 3) to end-of-treatment-FMD% (Visits 2 and 4) was a reduction of 0.7 ± 4.3% after apple extract and the corresponding change after placebo was a reduction of 0.1 ± 4.1%. The difference between the apple extract and placebo treatments was not statistically significant (*p* = 0.449). However, the sequence of treatments in this cross-over trial showed an effect with the sequence of apple extract-placebo being superior to the placebo- apple extract sequence (1.7%, CI from 0.2% to 3.2%, *p* = 0.029).

The findings were similar when the data were analyzed with FMD% calculated as the mean FMD% instead of the maximal FMD%. Mean FMD% was calculated as the mean of the artery diameter estimates at 40, 60 and 80 s after cuff release.

### Secondary efficacy parameters

#### NMD%

The NMD% averaged ~24% in all four measurements, with no statistically significant differences between the treatments in the change from the baseline (Visits 1 and 3) to the end-of-treament (Visits 2 and 4) (mean change 0.7%, CI from −0.4% to 2.0%, *p* = 0.301).

#### BP and biomarkers

Systolic BP did not change significantly during the Treament Periods. For diastolic BP, there was a significant sequence effect; the difference at end-of-treatment was −3.3 mmHg (ANCOVA *p* = 0.008, 95% CI from −5.6 to −0.9). There were no differences between the treatments in the biomarker concentrations. No significant correlations between acute FMD% and acute changes (pre to post Visit blood draw) in the individual biomarkers (sICAM-1, sVCAM-1, PAI-1, CRP, ADMA, vWf and sE-selectin) were found (Table 3).

**Table 3:** Acute change from baseline in blood pressure and biomarkers

	Apple extract treatment			Placebo treatment		
Variable	Change	Mean (SD), pre	Mean (SD), post	Change	Mean (SD), pre	Mean (SD), post
Systolic blood pressure, mm Hg^a^	−0.6	145.4 (13.2)	145.3 (15.5)	−1.6	148.2 (15.1)	146.7 (14.5)
Diastolic blood pressure, mm Hg^a^	−0.6	90.3 (7.1)	89.7 (8.0)	0.3	90.3 (7.0)	90.8 (7.1)
s-ICAM-1, pg/ml^b^	−8658	267,737 (52847)	259,086 (50958)	−5339	264,810 (58029)	259,375 (54260)
sVCAM-1, pg/ml^b^	−0.10 (log)	1,555,001 (520492)	1,415,276 (561821)	−0.07 (log)	1,494,420 (500826)	1,384,356 (422927)
PAI-1, pg/ml^b^	0.07 (log)	55,433 (28812)	61,956 (39638)	0.09 (log)	53,976 (27527)	58,703 (29755)
C-reactive protein, pg/ml^b^	−0.10 (log)	2,791,200 (9722821)	1,230,700 (1189128)	0.02 (log)	1,396,497 (2464468)	1,530,123 (2079646)
ADMA, μmol/lb	0.005	0.776 (0.175)	0.790 (0.144)	−0.012	0.791 (0.155)	0.780 (0.154)
vWf, ng/ml^b^	0.009 (log)	64,201 (40893)	71,662 (71789)	0.013 (log)	58,823 (40455)	65,056 (55217)
sE-selectin, pg/ml^b^	0.09 (log)	689.2 (315.4)	751.8 (295.6)	0.13 (log)	649.9 (279.8)	741.3 (301.3)

^a^Change from baseline at end-of-treatment period (based on ANCOVA-model)

^b^Change in pre-dose measurements between end and start of the treatment period (based on ANCOVA-model)

#### Epicatechin and its metabolites

Total epicatechin (including epicatechin, catechin, (epi)catechin sulpho-glucuronide, (epi)catechin glucuronide, methyl-(epi)catechin glucuronide, (epi)catechin sulphate, methyl-(epi)catechin sulphate, methyl-(epi)catechin, (epi)catechin disulphate and (epi)catechin diglucuronide) concentrations in blood increased significantly following acute consumption of apple extract compared to placebo (Table 4). Long-term changes (Pre Visit samples in Visits 1 and 3, compared to Pre Visit samples in Visits 2 and 4) in blood epicatechin concentrations were small and statistically non-significant.

**Table 4:** Change in total epicatechin and it’s metabolites concentration (μmol/L) from predose measurement within visit

	Apple extract		Placebo	
	First visit	Last visit	First visit	Last visit
N	58	56	59	58
Mean	2.474	2.873	0.004	0.001
SD	1.795	2.001	0.024	0.009
min	0.000	−0.004	−0.024	−0.030
max	7.144	8.391	0.167	0.058

min, minimum; max, maximum; SD, Standard Deviation

#### Safety

Adverse events (AEs) were reported similarly during the active and placebo administration (Table 5). The most common AE was headache, which usually occurred during the Visits after the NMD testing with isosorbide dinitrate. No clinically significant changes in the mean results of the laboratory safety assessments were detected (Table 6).

**Table 5:** Adverse events reported during apple extract and placebo administration in the study. There were no statistical diffrences between the groups

Description	Epicatechin N (%)	Placebo N (%)
Nasopharyngitis	5 (8.6)	3 (5.1)
Dyspepsia	2 (3.4)	2 (3.4)
Headache	15 (25.9)	14 (23.7)
Migraine	1 (1.7)	2 (3.4)
Dizziness	1 (1.7)	2 (3.4)
Ligament sprain	1 (1.7)	2 (3.4)
Gammaglutamyltransferase increased	1 (1.7)	2 (3.4)

**Table 6:** Change from baseline in main laboratory safety parameters, mean values

		Apple extract treatment		Placebo treatment	
Variable	Screening (SD)	Change	Mean (SD)	Change	Mean (SD)
Haemoglobin (g/l)	143.1 (11.5)	1.5	144.7 (1.5)	1.4	144.5 (1.4)
Haematocrit	0.425 (0.030)	0.001	0.426 (0.029)	0.003	0.427 (0.030)
Retikulocytes (E9/l)	1.24 (0.34)	0.06	1.31 (0.45)	0.02	1.26 (0.42)
Mean corpuscular volyme (fl)	88.8 (4.3)	0.1	88.9 (4.3)	0.2	89.0 (4.4)
Mean corpuscular haemoglobin (pg)	30.0 (1.8)	0.3	30.4 (2.0)	0.1	30.1 (1.9)
Leucocytes (E9/l)	4.92 (1.27)	−0.24	4.64 (1.13)	−0.11	4.77 (1.06)
Plasma albumin (g/l)	39.7 (2.5)	−0.8	38.9 (2.0)	−1.0	38.8 (2.1)
Plasma bilirubin (μmol/l)	11.6 (6.4)	−0.4	11.4 (6.8)	−0.8	11.1 (6.0)
Plasma alanine aminotransferase (U/l)	25.0 (14.8)	1.5	25.7 (13.9)	2.3	27.1 (14.4)
Plasma aspartate aminotransferase (U/l)	26.6 (8.4)	−1.5	24.8 (6.4)	−0.1	26.6 (10.4)
Plasma creatinine (μmol/l)	78.8 (12.7)	0.9	79.7 (12.6)	0.1	79.1 (12.5)
Plasma glutamyl transferase (U/l)	27.6 (31.3)	2.6	27.2 (33.9)	5.2	32.7 (60.9)
Plasma sodium (mmol/l)	141.3 (1.6)	−0.2	141.1 (1.7)	−0.3	141.0 (1.6)
Plasma potassium (mmol/l)	4.00 (0.31)	0.07	4.07 (0.26)	0.05	4.04 (0.27)
Fasting plasma calcium (mmol/l)	2.31 (0.08)	0.00	2.31 (0.08)	0.00	2.30 (0.08)
Fasting plasma urea (mmol/l)	5.18 (1.18)	−0.13	5.03 (1.04)	0.06	5.24 (1.21)
Plasma uric acid (μmol/l)	301.2 (72.4)	−1.5	298.1 (71.3)	−9.1	291.3 (69.3)
Fasting plasma total cholesterol (mmol/l)	5.65 (0.91)	−0.06	5.55 (1.02)	−0.09	5.56 (0.91)
Fasting plasma HDL-cholesterol (mmol/l)	1.88 (0.52)	−0.10	1.76 (0.49)	−0.09	1.78 (0.48)
Fasting plasma LDL-cholesterol (mmol/l)	3.27 (0.91)	0.01	3.26 (0.91)	0.02	3.29 (0.78)
Fasting plasma triglycerides (mmol/l)	1.10 (0.56)	0.04	1.14 (0.72)	−0.03	1.07 (0.52)
Fasting plasma glucose (mmol/l)	5.42 (0.46)	0.11	5.52 (0.48)	0.11	5.52 (0.49)
Plasma C-reactive protein (mg/l)	2.43 (1.57)	−0.53	1.86 (1.23)	1.71	3.75 (5.24)

#### Post-hoc analysis

Additional post-hoc analyses were conducted to evaluate the role of baseline FMD%, gender and age to the FMD response. After placebo and apple extract treatments, subjects with low baseline FMD% (<8 at the start of the treatment period) showed positive FMD responses and accordingly subjects with high FMD% (≥8 at the start of the period) responded negatively. No statistically significant effects of gender or age on the FMD responses were seen.

## Discussion

This randomized, double-blind, placebo-controlled cross-over study showed a significant acute improvement in FMD of the brachial artery, an indicator of endothelial function, in subjects with borderline or mild hypertension after intake of the epicatechin-containing study product. Nevertheless, when contrasted with placebo administration, the study did not reveal statistically significant improvements in FMD, neither associated with acute ingestion nor long-term consumption of the epicatechin product. In addition, no significant effects on the secondary efficacy variables, NMD, BP and biomarkers of vascular function, were found.

Endothelial function can be non-invasively assessed by ultrasound imaging. This ultrasound test measures flow-mediated changes in the diameter of the brachial artery. Flow-mediated changes in brachial artery diameter are caused by shear stress -induced generation of endothelial-derived vasoactive mediators. Since the brachial artery dilatation response to shear stress can be almost completely blocked by pretreatment with nitric oxide synthase inhibitors [23], it has been suggested that the phenomenon is predominantly due to endothelial release of nitric oxide. Increasing evidence suggests that brachial FMD is not only associated with traditional cardiovascular risk factors but also with future cardiovascular events [10, 24, 25]. A recent meta-analysis estimated that a 1 % decrease in FMD and a one SD decrease in FMD are associated with 8% and 22% increases in the risk of future cardiovascular events, respectively [26]. Risk factors that have been associated with impaired FMD responses include hypertension, increasing age, dyslipidemia, cigarette smoking, passive smoking, and obesity [9, 13, 27–29]. Dietary factors that have been shown to impair FMD responses include e.g. high intake of sodium [30] and, saturated fats [31], whereas the intake of vitamin C [32], vitamin D3 [33] and flavonoids [3–5] improve FMD responses.

Many flavonoids are potent antioxidants in in vitro systems. High intake of flavonoid-rich foods has been associated with reduced cardiovascular morbidity and mortality in humans [34–36]. In the current study, we found no significant improvement in endothelial function, as assessed with the FMD test, after ingestion of the dietary supplement epicatechin, a flavonoid enriched in a specific variety of apples, compared to placebo, although significant acute improvements were observed after epicatechin intake.

According to previous studies, the brachial FMD response is reduced in individuals with elevated BP compared to individuals with healthy BP levels [18]. Importantly, the inverse association between systolic BP and the brachial FMD response is linear. Elevated BP may interfere with the integrity of arterial endothelial cells, causing endothelial dysfunction [9]. In the present study that included individuals with elevated BP values, the FMD values averaged ~6-7%. As expected, these responses were smaller than those reported in the general Finnish population that are typically in the range of ~8-9% [37]. However, as no pre-selection of the study subjects was done based on the baseline FMD responses, many pre-hypertensive individuals with FMD values within the normal range were included in the study.

The primary aim of this study was to test the hypothesis that an orally ingested apple polyphenol extract rich in epicatechin and flavan-3-ol oligomers improves brachial artery endothelial function in volunteers with borderline hypertension or mild unmedicated hypertension, either acutely or in a longer term, or both. At treatment start, acute apple extract administration was associated with a mean FMD% increase of ~0.9%. During the end-of-treatment visit, the corresponding FMD% increase was ~1.5%. However, small acute improvements in FMD% were also seen after placebo intake. The corresponding changes after placebo were ~0.4 and ~0.7%. Therefore, the superiority of apple extract over placebo did not reach statistical significance. There is no clear explanation why the administration of placebo was associated with enhanced FMD responses. Mental stress has been associated with endothelial dysfunction [38, 39], and one possible explanation is alleviation of the initial stress related to the vascular testing situation in the course of the study visits, as the subjects became accustomed to the testing environment and circumstances.

The changes in FMD% after intake of apple extract showed large inter-individual variation, ranging from a small reduction of 1% to a maximum increase of 24.6%. With placebo, the corresponding minimum and maximum changes were −2.8% and 20.9%. Despite this large variation, the standard deviations for FMD% were quite similar to those reported earlier from a healthy Finnish population in a similar age range (*n* = 2185), where the mean FMD% was greater (8.9%) than that observed in the current study, but the standard deviation was of similar magnitude (SD 4.5) [40].

After four weeks of once-daily intake of apple extract, there was a sustained increase in epicatechin concentrations in plasma. Despite this, however, and the suggestive acute vascular effects, there was no significant improvement in FMD at four weeks. The reasons for this lack of efficacy are unclear, but may indicate some kind of desensitization to the vascular effects of epicatechin associated with prolonged exposure. One explanation could be that the subjects did not have significant enough hypertension for the effects of the extract to be significant. An analogous finding has been previously reported with vitamin C. In cigarette smokers, short-term oral administration of vitamin C acutely improved brachial FMD responses but there was no sustained effect after eight weeks of treatment [41].

Biomarkers of endothelial dysfunction and atherosclerosis were analyzed from blood samples collected at the start and end of both Treatment Periods. Apple extract supplementation did not have statistically significant effects on the levels of sICAM-1, sVCAM-1, ADMA, PAI-1, vWf, sE-selectin, or CRP. The mean levels of CRP, sICAM-1, and sE-selectin (Table 3) were well within the reference ranges found in the literature [42–44]. In contrast, the mean concentrations of total PAI-1, sVCAM-1, and ADMA (Table 3) in the study population were at the high end of the normal range, which is in line with the study population being borderline hypertensive. It is important to note, however, that the published reference ranges may not be transferrable to other populations, depending for example on different genetic backgrounds and environments. For total vWf protein concentration, reference ranges were not found in the literature, because in the clinical diagnostics, the biological activity of vWf is measured rather than the concentration of the protein. The statistical associations between the biomarkers and total epicatechin levels in blood were evaluated but no significant correlations were found. As the levels of the biomarkers were within or at the high end of the literature-based reference ranges (i.e. close to normal), it may be that the possible effects of epicatechin, if any, on pathological levels of these markers remained undetected. Also, statistically significant associations were not found between the levels of the biomarkers and acute FMD%, which is in line with the results above.

In a dietary intervention trial such as the present study, it is important to make an attempt to control for large fluctuations in diet. For this reason, the subjects were advised to maintain their normal diets during the duration of the study and not make any major changes. In addition, high intake of epicatechin was an exclusion criterion in this trial. According to food diaries, the macronutrient intakes were similar as those reported by national dietary intake statistics [45]. Furthermore, no differences in daily dietary intake of macronutrients, vitamins or major food groups known to contain flavonoids (e.g. apples, tea, cocoa, red wine) were seen between the baseline records or those of the intervention periods. The dietary habits as reported by the study subjects thus offer no explanation for the lack of efficacy of apple extract. The subjects came to the visits after fasting overnight and were offered a standard snack after the first blood samples were drawn. The snack included a bread-roll and sugar-free juice. In an earlier study by Järvisalo et al. [46], eating a low-fat breakfast or lunch did not have any significant effect on FMD, so it is unlikely that the snack offered in this study would explain the lack of difference between the apple extract and placebo interventions on FMD.

The reasons for the lack of a significant effect of apple extract on FMD compared to placebo in this study are unclear. Future trials should further evaluate determinants of efficacy, e.g. studying individual differences in epicatechin pharmacokinetics might offer information on the best timing of the FMD% measurement after administration of epicatechin when evaluating acute effects. In this study, FMD% was measured at 1½ hours after administration of the investigational product, but analysis of serum concentrations of epicatechin showed that there were some individuals whose epicatechin concentration in serum was still undetectable 2 h after apple extract administration. Also, it would be of interest to carry out a dose-finding study and to repeat an acute and long-term effect study by prescreening the study subjects and include only those who have been shown to efficiently absorb epicatechin.

The present study population included many individuals with FMD responses within the normal range in spite of elevated BP. Therefore, future studies could consider prescreening the study subjects to include only individuals with documented endothelial dysfunction, e.g. with FMD responses repeatedly below the population average (FMD% 8.9 ± 4.5, Raitakari, personal communication). In an attempt to increase statistical power, the current study employed a cross-over design instead of a parallel design. Despite this, the study may have been underpowered, and it is recommended that future studies should take into account the large variation in FMD responses when calculating statistical power. The study showed that the sequence of administration had significant effects on the FMD results which is a valid reason to recommend a parallel-group design in future studies. In the selection of a population with elevated BP, future studies might also consider the use of ambulatory BP measurements, in order to further refine the target population by exclusion of subjects with transient white-coat hypertension. This might lead to more accurate target group selection.

## Conclusions

We found a significant acute improvement in maximum FMD% with apple extract rich in epicatechin administration. However, superiority of apple extract over placebo was not statistically significant in our study subjects with borderline hypertension or mild hypertension, largely due to an unanticipated effect of increased FMD in response to the placebo capsules. The overall long-term effect of apple extract on FMD% was not different from placebo. Furthermore, no differences between the apple extract and placebo treatments were observed for endothelium-independent NMD, BP or biomarkers of vascular function. The study raised no safety concerns regarding the daily administration of 100 mg epicatechin in the form of this apple polyphenol extract rich in epicatechin and flavan-3-ol oligomers, which is in line with data from animal safety studies [47] and a human intervention where healthy adults consumed 1-2 g/day cocoa flavanols for a period of 6 weeks [48].

## Abbreviations

ADMA: Asymmetric dimethylarginine
BMI: Body mass index
BP: Blood pressure
CRP: C-reactive protein
eNOS: Nitric oxide synthase
FMD: Flow mediated dilatation; endothelial
HDL: High density lipoprotein
HR: Heart rate
LDL: Low density lipoprotein
NMD: Nitrate-mediated vasodilatation
PAI-1: Plasminogen activator inhibitor-1
SD: Standard deviation
sE-selectin: Soluble E-selectin
sICAM: Soluble intercellular adhesion molecule-1
sVCAM: Soluble vascular cellular adhesion molecule
vWF: Von Willebrand factor
